# Mesenchymal stem cell infiltration during neoplastic transformation of the human prostate

**DOI:** 10.18632/oncotarget.17362

**Published:** 2017-04-21

**Authors:** W. Nathaniel Brennen, Baohui Zhang, Ibrahim Kulac, L. Nelleke Kisteman, Lizamma Antony, Hao Wang, Alan K. Meeker, Angelo M. De Marzo, Isla P. Garraway, Samuel R. Denmeade, John T. Isaacs

**Affiliations:** ^1^ Department of Oncology at the Sidney Kimmel Comprehensive Cancer Center at Johns Hopkins, Baltimore, MD, USA; ^2^ Department of Urology, David Geffen School of Medicine at UCLA, Los Angeles, CA, USA; ^3^ Department of Pathology at the SKCCC at Johns Hopkins, Baltimore, MD, USA; ^4^ Department of Urology, James Buchanan Brady Urological Institute, Johns Hopkins University School of Medicine, Baltimore, MD, USA

**Keywords:** mesenchymal stem cells, prostate cancer, benign prostatic hyperplasia, stem/progenitor cell, tissue-specific stem cell

## Abstract

Mesenchymal Stem Cells (MSCs) have been identified in prostate cancer, raising the critical question of their physical and temporal source. Therefore, MSCs were quantified and characterized in benign and malignant prostate tissue representing different disease states and a wide range of age groups from fetal development through adult death using analytical and functional methodologies. In contrast to lineage-restricted Mesenchymal Progenitor Cells (MPCs) found in normal prostate tissue, MSCs with tri-lineage differentiation potential (adipogenesis, osteogenesis, and chondrogenesis) are identified in prostate tissue from a subset of men with prostate cancer, consistent with an influx of more stem-like progenitors (i.e. MSCs) from the bone marrow. Additionally, prostate tissue from a subset of these patients is highly enriched in MSCs, suggesting their enumeration may have prognostic value for identifying men with aggressive disease. This influx is an ongoing process continuing throughout disease progression as documented by the presence of MSCs in metastatic lesions from multiple organ sites harvested at the time of death in metastatic castration-resistant prostate cancer (mCRPC) patients. This infiltration of MSCs from systemic circulation provides the rationale for their use as a cell-based vector to deliver therapeutic agents.

## INTRODUCTION

The prostate has the highest rate of neoplastic transformation in the human body, affecting >90% of men by their 8^th^ decade of life when accounting for benign and malignant growth. Paradoxically, this is true despite having one of the lowest proliferative indexes of any tissue (<0.2%/day) [1]. This observation is true throughout the world, though relative ratios of benign to malignant transformation vary geographically, suggesting a complex interplay between intrinsic pathophysiological forces and external environmental factors that drive the manifested phenotype. Due to its anatomical location and function, the prostate serves as a conduit for concentrated sperm and urine [2], in addition to being directly connected with the external environment via the urethra. As a result, the prostate is continually assaulted with potentially inflammatory insults via internal and external routes of exposure, including infectious agents, dietary carcinogens, urinary reflux, hormonal changes, and physical trauma [3].

Consequently, as a result of these insults ≥80% of men have histologic evidence of inflammation found in prostate biopsy samples [3]. Furthermore, chronic inflammation has been implicated in the initiation and progression of benign prostatic hyperplasia (BPH) and prostate cancer [3, 4]. In addition to recruiting the more canonical cells of the innate and adaptive immune system, these inflammatory stimuli mobilize and attract mesenchymal stem cells (MSCs) from the bone marrow to sites of tissue damage where they contribute to repair via regenerative and immunosuppressive functions [5–7].

MSCs are multipotent cells functionally characterized by the ability to differentiate into cells of the mesoderm-lineage, including osteoblasts, chondrocytes, adipocytes, fibroblasts, and smooth muscle, among others [8]. MSCs are defined analytically by the co-expression of CD73, CD90, and CD105 in the absence of hematopoietic lineage markers, such as CD14, CD20, CD34, CD45, and HLA-DR [8]. In the adult, MSCs are reported to represent between 1 in 10,000 and 1 in 100,000 cells in the bone marrow, where they contribute to the hematopoietic stem cell (HSC) niche [9, 10]. Furthermore, they can be mobilized from these niches in response to inflammatory stimuli, such as CXCL12, CCL5, CCL2, and TGF-β, all of which are upregulated in prostate cancer [6, 11, 12].

We have previously demonstrated that MSCs are present in prostate tissue from men with primary prostate cancer [5, 13]. This raises the critical question of whether these MSCs are derived from endogenous local sources, potentially present during fetal development of the prostate, or represent an influx into the prostate from more distant reservoirs (i.e. the bone marrow) during adult aging in response to a combination of systemic and local chemotactic signals. Answering this question is critical because if MSCs continually infiltrate sites of prostate cancer, they can be used as a cell-based vector for targeted delivery of therapeutic agents [14]. Therefore, to resolve this important question, we used analytical and functional methods to quantify and characterize MSCs in human prostate tissue from fetal development to adult death. This information was then used to justify a first-in-man clinical trial to evaluate the magnitude of MSC homing to sites of prostate cancer following systemic injection.

## RESULTS

### Identification of MSCs during fetal development of the human prostate

**Figure 1:**
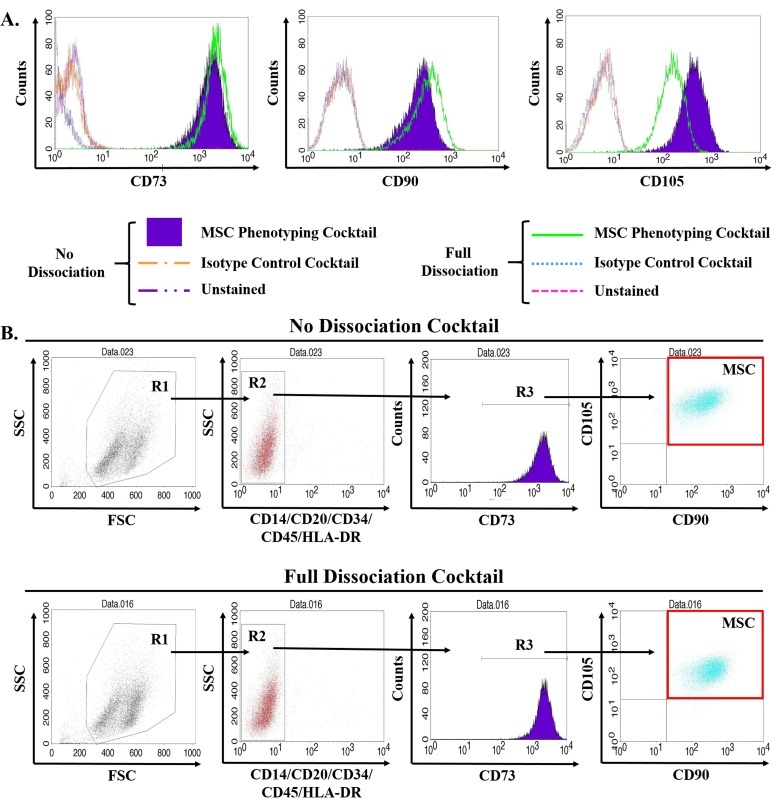
Validation of multiparameter flow cytometry assay to accurately quantify MSCs in primary human tissue MSCs are defined by multiparameter flow cytometry as the CD73, CD90, and CD105 triple-positive population that is negative for hematopoietic lineage markers (i.e. CD14, CD20, CD34, CD45, and HLA-DR). Primary human bone marrow-derived MSCs obtained commercially were expanded according to standard protocols, trypsinized, and incubated in the tissue dissociation cocktail for 1hr including three rounds of mechanical digestion using a gentleMACs dissociator prior to staining with antibodies for MSC phenotyping as previously described [5, 13]. **A**. Of the three canonical positive markers, only CD105 was significantly affected by the tissue dissociation protocol, but still remained strongly positive on these cells. **B**. Reduction in CD105 signal intensity did not impact quantification of MSCs. Digestion and analysis performed in triplicate with representative plots shown.

Due to the requirements for stromal cells during organogenesis, we hypothesized the developing fetal prostate would be a rich source of stromal progenitor cells, including MSCs. To assess this hypothesis, we optimized a multiparameter flow cytometry assay based on canonical positive (i.e. CD73, CD90, and CD105) and negative (i.e. CD14, CD20, CD34, CD45, and HLA-DR) MSC markers [5]. To provide further validation of this methodology, assay variability and epitope stability in the presence of the tissue dissociation cocktail were assessed using human bone marrow-derived MSCs (Figure 1). These analyses demonstrate the dissociation protocol does not impact MSC quantification and document a coefficient of variation (CV) of <0.003 (Table 1). Therefore, MSCs can be accurately and reproducibly quantified using the analytical assay described herein.

**Table 1:** Multiparameter flow cytometry assay used to quantify MSCs in primary human tissue is highly reproducible

Pre-analysis Incubation Conditions	Analytic Marker(s)	Replicates (% of 10,000 events)			Average (%)	Standard Deviation (SD)	Standard Error (SE)	Coefficient of Variation (CV)
		1	2	3				
**No Dissociation Cocktail**	**MSCs** **(triple-positive)**	97.1	97.5	97.0	97.2	0.3	0.2	0.0029
	**CD73**	97.8	98.1	97.6	97.8	0.2	0.2	0.0023
	**CD90**	97.1	97.5	97.0	97.2	0.3	0.2	0.0029
	**CD105**	97.8	98.1	98.1	97.8	0.2	0.2	0.0024
**Full Dissociation Cocktail**	**MSCs** **(triple-positive)**	98.7	98.7	98.7	98.7	0.0	0.0	0.0001
	**CD73**	98.9	99.0	98.7	99.0	0.0	0.0	0.0005
	**CD90**	98.9	98.9	98.9	98.9	0.0	0.0	0.0003
	**CD105**	98.7	98.8	98.7	98.7	0.1	0.0	0.0005

The experiment described in Figure 1 was performed in triplicate to determine intra-assay variability (i.e. reproducibility), which documented a coefficient of variation for the assay of <0.003

The prostate develops embryonically from the urogenital sinus (UGS), which is comprised of epithelial and mesenchymal components (i.e. UGE and UGM, respectively). The developing UGS was hypothesized to be a rich source of progenitor cells, including MSCs, due to the requirements of organogenesis. Indeed, using this assay, we demonstrate ~3% (median: 2.2%) of the cells in human UGS are consistent with an MSC phenotype (Table 2A). Consistent with the high replicative potential associated with a stem or progenitor phenotype, rapid enrichment of the MSC population is observed following expansion of UGM-derived stromal cells in tissue culture. In all seven independent cultures analyzed, the majority of cells (≥85% in 4/5 samples) are consistent with an MSC phenotype within just a few (≤3) passages (Table 2B). A similar phenomenon is observed using primary stromal cultures from adult prostate tissue [13].

Table 2Identification of MPCs in the developing human fetal prostate

**Table:** A. Quantification of MSCs in Fetal Prostate

Sample	Age (wks)	Percent MSCs (%)
**UGS-1**	16	2.24
**UGS-2**	16	4.43
**UGS-3**	17	1.97
**UGS-4**	17	3.81
**UGS-5**	20	1.84

**Table:** B. Rapid Selection of MSCs in Tissue Culture

Sample	Percent MSCs @ p3 (%)
**UGM-6c**	89.5
**UGM-7c**	56.2
**UGM-8c**	86.2
**UGM-9c**	89.5
**UGM-10c**	95.6
**UGM-11c**	96.0
**UGM-12c**	95.2

**Table:** C. Multipotent Differentiation

Sample	Adipocyte	Osteoblast	Chondrocyte
**UGS-6c**	-	+	+
**UGS-7c**	-	+	+
**UGS-8c**	-	+	+
**UGS-9c**	-	+	+
**UGS-10c**	-	+	+
**UGS-11c**	-	+	NT
**UGS-12c**	-	+	+
**UGS-13c**	-	+	+

(A) Percentage of cells in UGS defined as MSCs by multiparameter flow cytometry (mean: 2.9%; median: 2.2%). (B) Percent of cells defined as MSCs by flow cytometry at passage 3 in primary stromal cultures derived from human UGS under standard tissue culture conditions. (C) Multipotent differentiation potential of primary stromal cultures derived from UGS.

### Multipotent differentiation potential of stromal progenitors in the developing human prostate

Functionally, MSCs are characterized by their ability to differentiate into osteoblasts, chondrocytes, and adipocytes, among others depending on conditions and MSC source. This diversity of lineage potential results from MSCs giving rise to progeny, termed Mesenchymal Progenitor Cells (MPCs), which lose their multipotency and undergo lineage-restricted differentiation [15–18]. Stromal cultures derived from human UGS contain cells that can differentiate into osteoblasts and chondrocytes, but not adipocytes (Table 2C). To verify stromal cells fulfilling the characteristic marker profile of MSCs are unable to generate adipocytes, FACS was used to isolate the CD73/CD90/CD105 triple-positive and triple-negative populations from a pooled (n = 3) UGS sample (i.e. UGS-13c). Of note, only the triple-positive population attached to the plate and survived post-sorting. Expansion of this population maintained MSC marker expression and confirmed the lack of adipogenic differentiation potential (Table 2C), suggesting these are MPCs that have undergone lineage restriction in the developing male fetus.

### Identification of MSCs and MPCs in young adult prostates from organ donors

**Figure 2:**
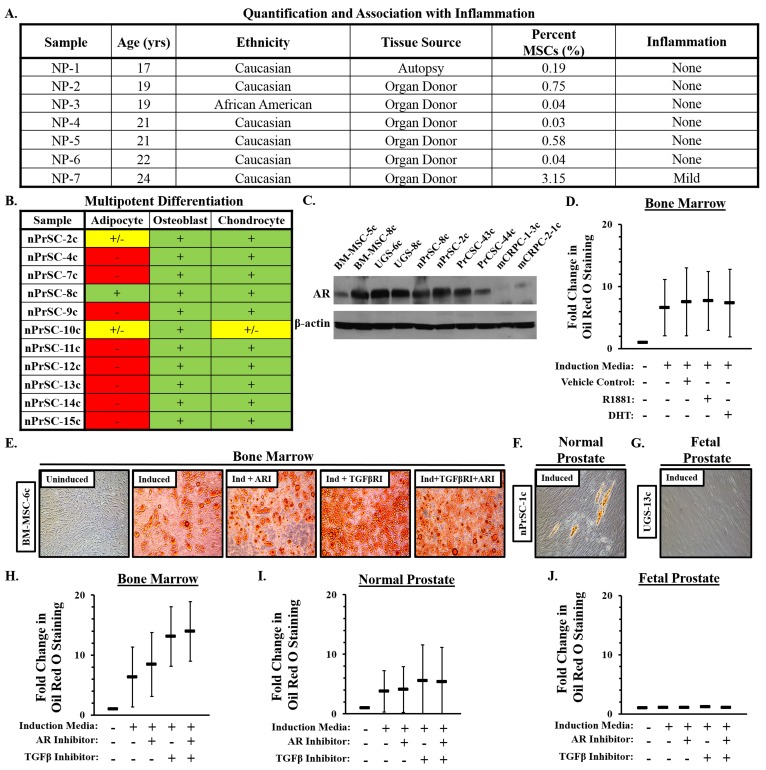
Characterization of mesenchymal stem and progenitor cells (MSCs/MPCs) in normal prostate tissue **A**. Percentage of cells defined as MSCs by flow cytometry in peripheral zone of prostates from young adult organ donors (mean: 0.7%; median: 0.2%). **B**. Multipotent differentiation potential of normal prostate stromal cultures. **C**. Androgen receptor (AR) expression in MSCs from bone marrow, fetal prostate, normal prostate, primary prostate cancer, and metastatic castration-resistant prostate cancer exposed to R1881 [1 nM, (18hrs)]. **D**. Adipogenesis in bone marrow-derived MSCs (n = 3) +/- R1881 (30nM), DHT (30nM), or vehicle (0.3% EtOH). **E**. Bone marrow-derived MSCs adipogenesis in the context of TGF-β inhibition [SB431542, (5μM)] +/- AR inhibition [Casodex, (20μM)]. **F**. Rare Oil Red O-positive cells or clusters of cells occasionally detected in a subset of normal prostate stromal cultures following adipogenic induciton. **G**. Oil Red O-positive cells were never observed in UGS-derived primary stromal cultures. Quantification of adipogenesis in **H**. bone marrow, **I**. normal prostate, and **J**. UGS stromal cultures. Three independent cultures of each analyzed in duplicate. Statistical analyses in Supplementary Tables 1, 2, 3, 4. Error bars: SD.

To determine whether MSCs and/or MPCs are also present in the mature prostate, we examined a series of prostates from young adults (<25 yo) obtained through a rapid organ donor program. A subset of cells in all samples (n = 7) were consistent with an MSC phenotype (Figure 2A). As expected, MSCs represent a relatively low percentage (~0.7%, median: 0.2%) of the overall cells present in the adult prostate. However, >3% of the cells in one case were defined as MSCs, a significantly higher number than the other cases. In contrast to the other samples in this age group, histologic examination revealed the presence of a mild chronic inflammatory infiltrate (Figure 2A), supporting the role of inflammation in mediating MSC recruitment. Like fetal prostate stromal cultures, those derived from the prostates of young adults are unable to undergo robust adipogenesis with a single exception (Figure 2B); indicative of lineage-restricted MPCs. Interestingly, a population of less committed progenitors (i.e. MSCs) that retain their adipogenic potential is present in a subset of young adults.

### TGF-β and AR regulate adipogenic differentiation potential in MSCs

The recruitment of MSCs as a function of inflammation raises the question of their fate within the prostate over time. Evidence suggests androgen signaling suppresses MSC adipogenesis [19–21]. Consistent with this observation, bone marrow- and prostate-derived stromal cells express the androgen receptor [AR, (Figure 2C)]. However, in contrast to previously reported data, differentiation of bone marrow-derived MSCs in the presence of dihydrotestosterone (DHT) or R1881, a highly potent non-aromatizable synthetic androgen, is unable to inhibit adipogenesis under the conditions tested (Figure 2D, Supplementary Table 1).

TGF-β also suppresses adipogenesis via downstream inhibition of PPARγ [22–24]. Inhibition of TGF-β signaling via SB431542 significantly stimulates adipogenesis in bone marrow-derived MSCs (Figure 2E, 2H, Supplementary Table 2). Inhibition of AR signaling via casodex has a small but significant additive effect when combined with TGF-β inhibition in these cells (Figure 2H, Supplementary Tables 3, 4). Very rare Oil Red O-positive cells or clusters of cells (Figure 2F) are detected in a subset of normal prostate stromal cultures following adipogenic induction (Figure 2I). However, this level of differentiation is typically below the level of sensitivity for quantification with one notable exception (Figure 2F, 2I, Supplementary Table 2). In contrast, no Oil Red O-positive cells are observed in any of the fetal prostate cultures even in the context of AR and/or TGF-β inhibition (Figure 1G, 1J, Supplementary Tables 2-4), suggesting lineage restriction is not easily reversible once fully programmed.

**Table 3:** MSCs are present in primary prostate cancer tissue independent of inflammation status

Sample	Age (yrs)	% MSCs	Gleason Score	Inflammation		
				Cancer	Benign	Atrophy
PCa-1	69	1.10	3+4	Mild	None	Moderate, Non-Focal
PCa-2	70	1.06	4+5	None	Mild	-
PCa-3	66	1.03	3+4	-	Moderate, Non-Focal	-
PCa-4	55	0.98	3+4	None	Mild	-
PCa-5	60	0.45	3+3	-	Mild	Mild
PCa-6	70	0.38	3+3	Mild	Mild	-
PCa-7	65	0.38	4+5	N/A	N/A	N/A
PCa-8	63	0.28	4+5	None	Mild	None
PCa-9	68	0.28	3+4	None	Mild	Mild
PCa-10	70	0.25	3+4	Mild	Mild	None
PCa-11	59	0.23	4+5	Mild	Mild	Mild
PCa-12	51	0.22	3+3	None	Mild	-
PCa-13	58	0.22	3+3	-	Acute, Mild	None
PCa-14	70	0.21	5+4	-	None	None
PCa-15	55	0.19	4+3	Mild	Moderate	Mild
PCa-16	65	0.14	4+3	None	None	None
PCa-17	61	0.08	3+4	Mild	None	None
PCa-18	70	0.08	4+3	Mild	Chronic, Mild, Non-Focal	None
				-	Acute, Moderate	-
PCa-19	57	0.07	4+5	None	None	None
PCa-20	54	0.06	4+3	-	None	-
PCa-21	59	0.06	4+5	Mild, Non-Focal	Mild	Mild
PCa-22	64	0.06	4+3	None	None	None
PCa-23	59	0.02	4+5	-	None	Mild
PCa-24	57	0.01	3+4	Mild	None	-
PCa-25	73	0.01	3+4	-	None	Mild
PCa-26	70	<0.01	3+4	None	None	None
PCa-27	63	<0.01	4+3	Moderate, Non-Focal	Mild, Non-Focal	None
PCa-28	49	<0.01	4+4	None	None	-
PCa-29	53	<0.01	4+3	None	Mild	-
PCa-30	62	<0.01	4+5	None	Mild	-
PCa-31	68	<0.01	3+4	-	Mild	Mild

Percent of cells defined as MSCs in radical prostatectomy tissue from men with prostate cancer by flow cytometry (mean: 0.3%; median: 0.1%). No association between the number of MSCs detected and the level of inflammation present in the tissue is observed. Benign, atrophic, and malignant areas were scored where appropriate for the type (chronic or acute) and intensity (none, mild, moderate, or severe) of inflammation present and whether it was focal or non-focal. Unless otherwise noted, all inflammation observed was chronic and focal. Notes: Tissue was unavailable for IHC analysis in PCa-7. PCa-18 had areas of chronic and acute inflammation. ‘-’ denotes not present in this sample

Table 4MSCs are present in metastatic castration-resistant prostate cancer (mCRPC) lesions from multiple organ sites at the time of death

**Table:** A. Quantification of MSCs in Metastatic Castration-Resistant Prostate Cancer

Sample	Age (yrs)	Tissue	% MSC
mCRPC-1-1	66	Liver	0.26
mCRPC-1-2		Liver	0.09
mCRPC-1-3		Lymph Node	0.06
mCRPC-2-1	57	Bone	0.15
mCRPC-2-2		Liver	<0.001
mCRPC-2-3		Lung	<0.001
mCRPC-3-1	81	Skin	0.003
mCRPC-3-2		Lung	<0.001
mCRPC-3-3		Lung	<0.001
mCRPC-3-4		Lymph Node	<0.001

**Table:** B. Multipotent Differentiation

Sample	Tissue	Adipocyte	Osteoblast	Chondrocyte
mCRPC-1-3c	Lymph Node	+	+	+
mCRPC-2-1c	Bone	+	+	+

(A) Percent of cells defined as MSCs by flow cytometry in metastatic lesions from mCRPC patients undergoing a rapid autopsy at the time of death (mean: 0.1%; median: 0.002%). MSCs could be detected in 50% of mCRPC lesions from distinct organ sites. (B) MSCs expanded from mCRPC lesions obtained from distinct organ sites in independent patients at the time of death have tri-lineage differentiation potential (i.e. adipogenesis, osteogenesis, and chondrogenesis).

### Incorporation of stromal progenitor cells from systemic sources into prostate smooth muscle

**Figure 3:**
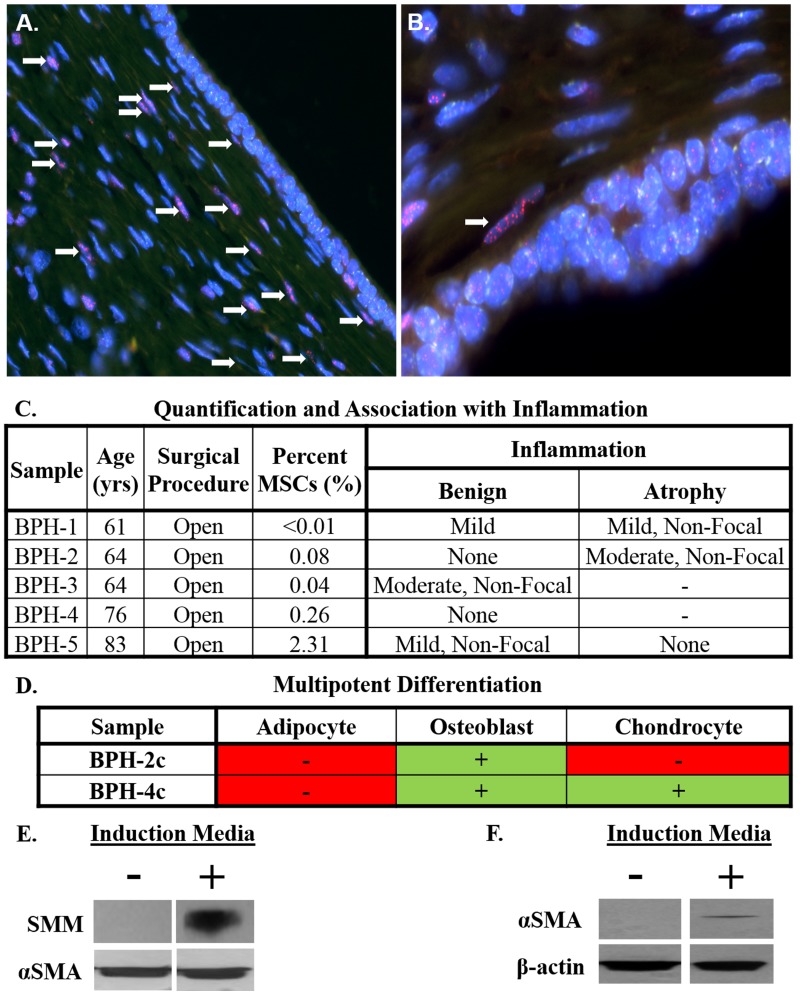
Stromal progenitors infiltrate prostate tissue and undergo smooth muscle differentiation, potentially contributing to benign prostatic hyperplasia (BPH) pathogenesis in the context of chronic inflammation **A**. Human UGS implanted en bloc (i.e. undigested) under the renal capsule of nude rats [25]. Infiltrating rat stromal cells incorporate into the peri-glandular smooth muscle layer (white arrows) as demonstrated by dual-labeling with FISH probes specific for telomeres (red) and human centromeres (green) to differentiate between rodent (red) and human (green) cells [26]. Nuclei stained with DAPI (blue). 200x magnification. **B**. 400x magnification. **C**. Percentage of cells defined as MSCs by flow cytometry in open prostatectomy tissue from men with symptomatic BPH (mean: 0.5%; median: 0.1%). Tissue scored for the type of inflammation (acute or chronic), intensity (mild, moderate, or severe), and whether it was focal or non-focal in benign and atrophic areas when present. Unless otherwise noted, inflammation was chronic and focal in nature. **D**. Multipotent differentiation potential of primary stromal cultures from open prostatectomy tissue. **E**. Upregulation of smooth muscle myosin (SMM) in rat bone marrow-derived MSCs co-cultured with rat bladder smooth muscle cells in the presence of sodium butyrate (1 mM) in a Transwell assay. **F**. Upregulation of alpha-smooth muscle actin (αSMA) in human bone marrow-derived MSCs co-cultured with human prostate smooth muscle cells in the presence of sodium butyrate (1 mM) in a Transwell assay.

The characteristic stromal hyperplasia observed in BPH suggests the presence of stromal progenitors. To evaluate whether MSCs from systemic sources invade prostate tissue and undergo smooth muscle differentiation, human fetal UGS implanted en bloc (i.e. undigested tissue including epithelium and stroma) under the renal capsule of nude rats [25]. Using a dual-label telomere and centromere fluorescence in situ hybridization (FISH) assay that can unambiguously distinguish between human and rodent cells in tissue recombinants [26], infiltrating rat cells identified by their long telomeres (bright red) and lack of human-specific centromere staining (green) can be found in the peri-glandular smooth muscle layer of the developing glands (Figure 3A, 3B). Though rodent cells do not make up the majority of the stroma, it's important to note the UGS was grafted en bloc complete with all of the endogenous fetal human MSCs and stromal cells present at the time of collection. Significantly, however, this clearly demonstrates that stromal progenitors are recruited from systemic sources into grafted prostate tissue and subsequently incorporate into the smooth muscle layer. Notably, the percentage of stromal cells derived from systemic sources remains relatively fixed (~15-20%) from day 30 to day 200 post-implantation despite the graft increasing in size by ~16-fold over this same period [25], suggesting that MSC recruitment is an ongoing process required for tissue growth and neoplastic expansion.

### Identification of MPCs in benign prostatic hyperplasia (BPH) tissue

Based on these results, we hypothesized that stromal progenitors could also be detected in the prostates of older men undergoing open prostatectomy for symptomatic BPH. Based on the analytical assay, MSCs represent ~0.5% (median: 0.08) of all cells present (Figure 3C). Again, the majority of cases have a fairly low frequency of MSCs with the exception of a single case (>2%), which had histologic evidence of non-focal mild chronic inflammation. In contrast to prostate tissue from young men, inflammation was commonly observed in the other samples within this group, including two with a more intense infiltrate.

### Paracrine factors in tissue microenvironment induce smooth muscle differentiation of MSCs

Similar to stromal cultures from fetal and young adult prostates, those from BPH tissue have undergone lineage restriction in response to paracrine factors in the microenvironment (Figure 3D). In support, rat bone marrow-derived MSCs undergo smooth muscle differentiation following co-culture with rat bladder smooth muscle cells in a Transwell system [27], which we have independently confirmed via upregulation of smooth muscle myosin [SMM, (Figure 3E)]. Of note, smooth muscle differentiation is enhanced by pretreating MSCs with sodium butyrate, a pan-histone deacetylase (HDAC) inhibitor. This is physiologically relevant because chronic inflammation induces epigenetic alterations in adjacent cells [28–30]. Importantly, we have extended these observations into a human model as demonstrated by the upregulation of alpha-smooth muscle actin (αSMA) in human bone marrow-derived MSCs following co-culture with very early passage prostate smooth muscle cells (Figure 3F). It should be noted these results could also indicate differentiation into pericytes or reactive fibroblasts.

### Identification of MSC/MPCs in primary and metastatic prostate cancer

**Figure 4:**
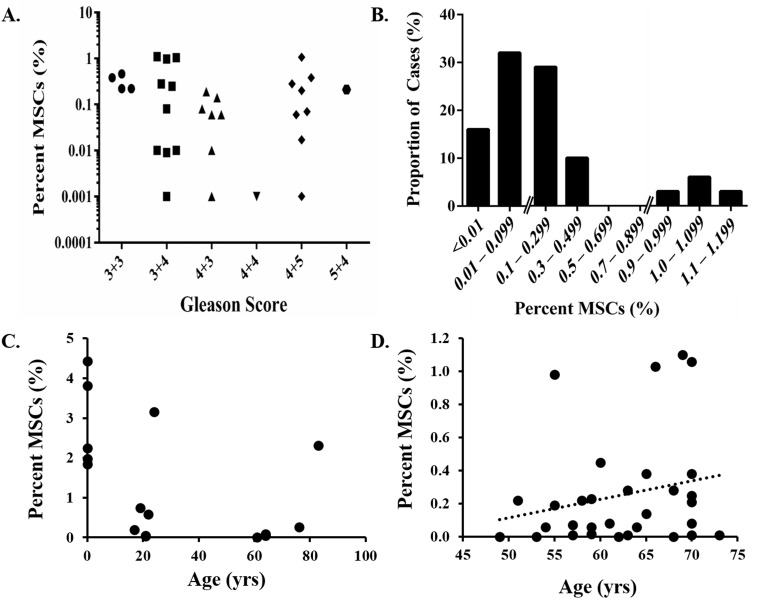
Quantification of MSCs in prostate cancer **A**. No association between MSC infiltration into prostate tissue and Gleason score. **B**. Bimodal distribution with a subset of cases (12%) highly enriched in MSCs (~1%). **C**. MSCs/MPCs in the prostate decrease as a function of age. However, a subset of the men in each of these groups has elevated numbers of MSC/MPCs. **D**. A trend towards increased numbers of MSCs/MPCs in the prostate with age is observed in men with primary prostate cancer.

We previously reported that MSC/MPCs are present in the prostates of men with prostate cancer [n = 10, [5]]. The current study extends those observations into >30 cases. This analysis demonstrated that MSC/MPCs represent ~0.3% (median: 0.1%, range: <0.001 – 1.10%) of cells in the peripheral zone of radical prostatectomy tissue (Table 3). No association with Gleason score is observed (Figure 4A). However, in contrast to the expected Gaussian distribution, a bimodal pattern is observed with the majority of cases having <0.5% and a subset of cases (12%) enriched in MSC/MPCs [~1%, (Figure 4B)]. Of note, only 1 of 10 high grade cancers (Gleason ≥8) in this series were in the enriched MSC fraction. MSC/MPCs were also detected in 5 of 10 metastatic lesions analyzed from 3 patients with lethal castration-resistant prostate cancer (mCRPC) at time of death (Table 4A). Multiple lesions from distinct organ sites (i.e. liver, bone, lymph nodes, lung, and skin) were analyzed from each patient with MSC/MPCs representing ~0.1% of the cells in these lesions.

### MSC/MPCs in the prostate decrease with age except under inflammatory or pathologic conditions

The frequency of MSCs in the bone marrow decreases with age [31]. Like the bone marrow, MSC/MPCs in the prostate also decline as a function of age from ~3% of the cells in human fetal UGS to ~0.7% in young adults and ~0.5% or less in older adults (Figure 4C). However, there is substantial heterogeneity within each of these groups with a subset of adult men having significantly elevated numbers of MSC/MPCs (≥1%) relative to their peers. Interestingly, there is a trend towards increased numbers of MSC/MPCs with age in men with primary prostate cancer (Figure 4D), suggesting a general enrichment over time potentially as a function of disease progression.

### No association between infiltrating MSC/MPCs and inflammation in malignant prostates

To determine whether the frequency of MSC/MPCs in radical prostatectomy samples is simply a function of the level of inflammation present in the tissue, a blinded analysis of 5 random cores from each case was performed by a urologic pathologist. No association between MSCs and inflammation is detected (Table 3). Inflammation is most frequently detected in benign areas of the tissue sections, though it is commonly present in atrophic and malignant areas as well (63%, 45%, and 45% of evaluable cases, respectively). Additionally, the inflammation observed is typically chronic, focal, and mild in nature. This suggests the relationship between inflammation and MSC infiltration is more complex in the context of cancer with the immunosuppressive properties of MSCs potentially leading to an inverse association.

### MSCs from a subset of prostate cancer patients retain adipogenic differentiation potential

**Figure 5:**
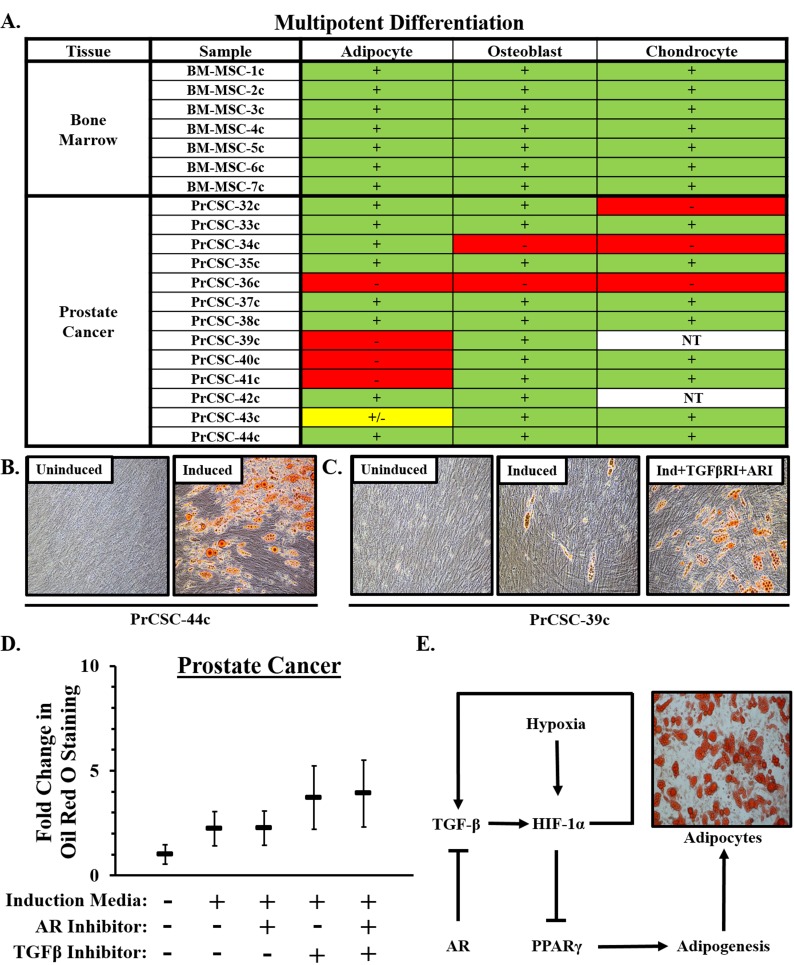
Multipotent differentiation of MSCs in prostate cancer **A**. Multipotent differentiation potential of bone marrow-derived MSCs and those from men with prostate cancer. NT = Not Tested. **B**. Representative pictures depicting robust adipogenesis of prostate cancer-derived MSCs when cultured under the appropriate induction conditions. **C**. Adipogenic differentiation potential was heterogeneous between cultures derived from independent donors, but consistently enhanced in the context of TGF-β inhibition [SB431542, (5μM)] with additive effects observed in combination with AR inhibition [Casodex, (20μM)]. **D**. Quantification of adipogenesis. Adipogenic potential of six independent cultures was evaluated in duplicate. Statistical analyses in Supplementary Tables 2, 3, 4. Error bars = SD. **E**. Schematic diagram of pathways regulating adipogenesis in MSCs showing convergence of TGF-β and AR signaling pathways on HIF-1α.

In contrast to lineage-restricted MPCs from fetal and young adult prostates, a subset of stromal cultures from prostate cancer patients retain tri-lineage differentiation potential (i.e. adipocytes, osteoblasts, and chondrocytes); a defining characteristic of bone marrow-derived MSCs (Figure 5A). Of note, there is significant inter-patient heterogeneity in the adipogenic response with some cultures undergoing robust differentiation (Figure 5B) and others doing so less efficiently (Figure 5C). The broader differentiation potential of MSCs from a subset of patients suggests the recruitment of less committed stromal progenitors (i.e. MSCs) from the bone marrow as a function of disease progression. Additionally, primary stromal cultures from metastatic lesions from distinct organ sites (i.e. bone and lymph node) obtained from independent mCRPC patients also displayed tri-lineage differentiation potential (Table 4B).

Also consistent with bone marrow-derived MSCs (Figure 2E), inhibition of TGF-β activity enhances adipogenesis in prostate cancer-derived MSCs (Figure 5C, 5E, Supplementary Table 1) with AR inhibition having a small additive effect in this context (Figure 5D, Supplementary Tables 3, 4). Interestingly, TGF-β inhibition restores adipogenesis in a subset of cultures that seemed to have lost this differentiation potential, suggesting lineage restriction may be reversible during the early stages of epigenetic programming. Oxygen tension is also a known regulator of stem cell biology with low oxygen concentrations (i.e. 1% O_2_, which is comparable to that present in the bone marrow microenvironment) [32] promoting a more ‘stem-like’ state [33–35]. We and others have previously demonstrated that hypoxia suppresses adipocyte differentiation [13, 36–38], suggesting that HIF-1α represents a point of convergence in these signaling pathways (Figure 5E).

## DISCUSSION

Our previous studies demonstrating MSCs are present in prostate cancer tissue raised the critical question of their physical and temporal source. Specifically, are they derived from endogenous local sources, potentially present since fetal development of the prostate, or do they represent an influx from more distant reservoirs (i.e. the bone marrow) into the prostate in response to a combination of systemic and local chemotactic signals during adult aging? Therefore, we examined benign and malignant prostate tissue from multiple donors representing different disease states and a wide range of age groups from fetal development through adult death using analytical and functional methodologies. This systematic analysis demonstrated MSCs from a subset of prostate cancer patients retain tri-lineage differentiation potential (i.e. adipogenesis, osteogenesis, and chondrogenesis), consistent with an influx of less committed (i.e. more stem-like) progenitors from the bone marrow. Thus, the conclusion from the present studies is that MSC recruitment is an ongoing process continuing throughout disease progression as documented by the presence of MSCs in metastatic lesions from multiple organ sites at the time of death in mCRPC patients.

Like the bone marrow [31], MSCs in normal prostate tissue decline as a function of age. However, a subset of adult men have elevated numbers of MSCs in their prostates (≥1%). This enriched fraction cannot be accounted for solely by peripheral blood contamination in the collected tissue since MSCs represent <0.03% of mononuclear cells in the peripheral blood and are frequently undetectable in healthy individuals [39, 40]. Interestingly, the frequency of MSCs in prostates from men with prostate cancer does not correlate with Gleason score, nor does it follow the expected Gaussian distribution. Rather, a bimodal pattern is observed with ~10-15% of cases containing elevated numbers of MSCs. Of note, this is approximately the same proportion of patients diagnosed with prostate cancer that eventually progress to lethal disease, suggesting that profiling MSCs in the tumor microenvironment may provide prognostic information independent of Gleason score. This hypothesis is supported by preclinical models demonstrating recruitment of MSCs to prostate cancer promotes metastasis through a CXCL16/CXCL12-dependent mechanism [41]. An association with disease progression may also be related to their immunomodulatory and pro-angiogenic properties [6, 7].

It should be noted that MSCs defined by the current criteria are still a heterogeneous population of cells, and MSCs identified in diverse tissue sources based on these markers may not be intrinsically or functionally similar. Indeed, MSCs are likely programmed by specific contextual signals within the tissue microenvironment to restrict function and differentiation. This is supported by data reported herein in which adipogenesis is suppressed via TGF-β and AR signaling pathways. Notably, these same pathways are also important for smooth muscle differentiation of MSCs [42–44], and the presence of an ARE in the TGF-β promoter provides evidence of coordinated crosstalk [45]. Together, these data suggest that MSCs recruited to the prostate lose their adipogenic potential as a function of committing to the smooth muscle lineage, which has noteworthy implications for the pathogenesis of BPH.

Additionally, the loss of lineage commitment in MSCs recruited to sites of prostate cancer indicates that tissue-specific ‘programming’ is disrupted due to an altered microenvironment. Provocatively, abnormalities in this lineage commitment may play a central role in prostate cancer pathogenesis as TGF-β is a well-known inducer of the reactive fibroblast or carcinoma-associated fibroblast (CAF) phenotype [46, 47]. CAFs promote tumor progression via multiple mechanisms [48], and their endogenous source has been the subject of intense study. Bone marrow transplant studies suggest that 20-40% of CAFs are derived from the bone marrow [49, 50], which represents a rich source of MSCs that can differentiate into CAFs [51]. Our data demonstrates that MSCs/MPCs are already present in the prostate during fetal development and represent a subset of cells in the adult prostate, suggesting that a larger percentage of CAFs may be derived from MSCs than previously recognized if one accounts for local and distant sources.

This recruitment of MSCs to the tumor microenvironment via systemic circulation suggests the potential to exploit this tumor tropism for cell-based delivery of therapeutic or imaging agents [5, 6]. This potential is made even more attractive by the ‘immuno-evasive’ properties of MSCs [52], which allow allogeneic MSCs to be used as an ‘off-the-shelf’ therapy for drug delivery without the need for HLA matching. Multiple strategies to develop MSCs as a tumor-targeting vector for advanced prostate cancer using microparticle [14] and genetic engineering platforms are currently in development by our multi-institutional, multi-disciplinary research team. Indeed, a phase 0 pre-prostatectomy clinical trial (NCT01983709) has already been initiated to quantify trafficking of allogeneic MSCs to sites of primary prostate cancer and demonstrate safety. As IV-infused MSCs will also traffic to non-tumor tissues throughout the body, a prodrug strategy such as those engineered to be selectively activated by tissue- or tumor-specific proteases, such as prostate-specific antigen (PSA), will need to be exploited to prevent toxicity to non-target peripheral tissues [14, 53–56]. It should also be noted there is no evidence of significant long-term engraftment or ectopic tissue formation in the thousands of patients that have been infused with allogeneic MSCs in hundreds of clinical trials worldwide for a variety of indications [52, 57], suggesting the risk of transformation or contributing to disease progression is exceedingly low.

## CONCLUSIONS

MSCs and/or MPCs have been identified in prostate tissue from its earliest embryonic origins through adulthood under normal and pathologic conditions ranging from BPH to lethal mCRPC. These cells have significant immunomodulatory properties and may play a role in tumorigenesis through several mechanisms including evading immunosurveillance, promoting angiogenesis, and generating CAFs, among others. Interestingly, MSCs with tri-lineage differentiation potential can be identified in a subset of prostate cancer patients, suggesting an influx of less committed progenitors from the bone marrow during cancer progression. This infiltration of MSCs into the prostate from systemic circulation provides the rationale for their use as a cell-based vector for the delivery of therapeutic or imaging agents. Additionally, prostate tissue from a subset of primary prostate cancer patients is highly enriched in MSCs, which does not correlate with inflammation or Gleason score, suggesting that enumeration of MSCs may have prognostic value for identifying men with aggressive disease.

## MATERIALS AND METHODS

### Reagents

Roswell Park Memorial Institute (RPMI)-1640 medium, keratinocyte-serum free medium (K-SFM), Dulbecco's Modified Eagle Medium (DMEM)/F12, DMEM (High Glucose), Hank's Balanced Salt Solution (HBSS), L-glutamine, and penicillin-streptomycin were purchased from Life Technologies-Invitrogen (Carlsbad, CA). Fetal bovine serum (FBS) was purchased from Gemini Bioproducts (West Sacramento, CA). hMSC high performance media was purchased from RoosterBio (Frederick, MD). Adipogenic, osteogenic, and chondrogenic induction media was purchased from Lonza (Walkerville, MD). The AR (N20) antibody was purchased from Santa Cruz (Dallas, TX). The β-actin antibody and SB431542 were purchased from Sigma-Aldrich (St. Louis, MO). DHT was purchased from Steraloids, Inc. (Newport, RI). R1881 was purchased from Perkin-Elmer (Boston, MA). Casodex was purchased from LKT Laboratories (St. Paul, MN).

### Primary tissue sources and cell culture

All tissues were collected in accordance with institutional review board (IRB)-approved protocols at the respective institutions. Fetal prostate samples (~14-18 weeks gestation) were obtained in accordance with federal and state guidelines, and prostate tissue was used for frozen/paraffin blocks or dissociated in order to obtain single cell suspensions and cultured as previously described [58]. A total of 13 fetal samples were used in this analysis. Bone marrow-derived MSCs (BM-MSC) were purchased from Lonza (BM-MSC-1c) or Rooster Bio (BM-MSC-5-8c; Frederick, MD). All other BM-MSC samples were obtained from healthy bone marrow donors through the Biospecimen Repository Core at Johns Hopkins in accordance with an IRB-approved protocol. Prostate tissue from young men (<25 yo; NP-1 to -8) was obtained through a rapid organ donor program organized by the National Disease Research Interchange (NDRI) in accordance with an IRB-approved protocol. Tissues were perfused, surgically harvested, and delivered within 24hrs of the time of death consistent with standard organ donor protocols to maximize viability. Tissue was then dissociated for analysis by flow cytometry or to generate primary stromal cultures as described below. BPH and prostate cancer tissue were obtained from patients undergoing an open or radical prostatectomy, respectively, at the Brady Urological Institute at Johns Hopkins via the Prostate Biospecimen Repository in accordance with IRB-approved protocols as previously described [5]. Metastatic prostate cancer was obtained from mCRPC patients at the time of death who had previously been consented into the rapid autopsy program at Johns Hopkins in accordance with an IRB-approved protocol. Normal prostate stromal cells (nPrSC)-9c were purchased from Lonza [i.e. Clonectics Prostate Stromal Cells (PrSC) from a 20yo donor], and the remaining normal prostate cultures (nPrSC-10c through -15c) were obtained as previously described [59].

Briefly, fifty 18-gauge biopsy needle cores (Angiotech, Vancouver, BC, Canada) were obtained from either open prostatectomy tissue in the case of BPH tissue or from the peripheral zone of radical prostatectomy tissue for prostate cancer specimens and washed in HBSS. Five randomly selected cores were fixed, paraffin-embedded, and sectioned or H&E staining and pathological evaluation. The remaining cores were mechanically minced and then enzymatically digested using a human tumor dissociation kit (Miltenyi Biotec, Inc., Bergisch Gladbach, Germany) and a gentleMACS dissociator (Miltenyi) according to the manufacturer's instructions. The dissociated cell suspension was then passed through a 70 μm pre-separation filter (Miltenyi), centrifuged at 2500 rpm for 5 min. The single cell suspension was resuspended in MACS cell sorting buffer (Miltenyi) to determine cell number and viability by trypan exclusion using a Cellometer Auto T4 (Nexcelcom Bioscience, Lawrence, MA). Importantly, this dissociation protocol is completed within 1 hr; thereby, minimizing artifacts associated with long exposure to digestive enzymes and prolonged ex vivo incubation. Cells were then analyzed by flow cytometry or plated in a T25 flask for expansion in tissue culture to generate nPrSC or prostate cancer stromal cell (PrCSC) cultures depending on the tissue source. All stromal cells were cultured in Rooster High Performance Media or RPMI-1640 medium supplemented with 10% FBS, 1% L-glutamine, and 1% penicillin-streptomycin in a 5% CO_2_, 95% air humidified incubator at 37°C with regular media changes every 3-4 days as previously described [13]. Primary epithelial cells (PrECs) were obtained and cultured in low-calcium serum-free defined media as previously described [60].

### Quantification of MSCs by flow cytometry

MSCs in primary tissue or culture were quantified as previously described [5, 13]. Briefly, cells were labeled with a MSC Phenotyping Cocktail (anti-CD14 PerCP, anti-CD20-PerCP, anti-CD34-PerCP, anti-CD45-PerCP, anti-CD73-APC, anti-CD90-FITC, and anti-CD105-PE) or Isotype Control Cocktail (Mouse IgG1-FITC, Mouse IgG1-PE, Mouse IGG1-APC, Mouse IgG1-PerCP, and Mouse IgG2a-PerCP) included in the human MSC Phenotyping kit (Miltenyi) in a volume of 100 μl per 1×10^6^ cells for 10m at 4°C according to the manufacturer's instructions. Anti-HLA-DR-PerCP (Miltenyi) was also added to the MSC Phenotyping Cocktail. Analysis was performed using a BD FACSCalibur flow cytometer. All compensation controls were performed using anti-EpCAM antibodies directly conjugated to FITC, PE, APC, or Biotin followed by anti-Biotin-PerCP on aliquots of the same cell suspension to ensure proper gating and instrument settings.

### Tissue recombination

*In vivo* tissue recombination studies was performed as previously described in accordance with Institutional Animal Care and Use Committee (IACUC)-approved protocols [26, 58]. Briefly, human fetal prostate stromal cells were co-inoculated in 50% matrigel with hTert-immortalized human prostate epithelial cells (PrECs) overexpressing c-Myc and the AR [60] at a 2:1 ratio (3×10^5^ total cells) subcutaneously into immune-deficient male NOD/SCID/gamma-null (NOG) mice (6-8 wks old) previously implanted with a time-release testosterone capsule. At 4 months post-inoculation, animals were euthanized by CO_2_ overdose, and grafts were harvested, weighed, fixed, and processed for immunohistochemical analysis as previously described [5, 54].

### Immunohistochemical staining

Immunohistochemical and immunofluorescent staining was performed by the SKCCC Immunohistochemistry Core as previously described [54]. Images were taken using a Nikon Eclipse Ti Fluoresecent scope equipped with a Nikon DS-Qi1 Mc camera and NIS-Elements AR3.0 imaging software.

### Fluorescence *in situ* hybridization (FISH)

Tissue sections of human fetal prostate tissue surgically implanted en bloc into the subrenal capsule of nude rats at 200-days post-implantation were generously provided by K. Boekelheide [25]. Dual-label human centromere-specific and telomere-specific FISH was performed to differentiate between rodent and human cells as previously described [26].

### Analysis of inflammation

Five random needle biopsy cores from each case harvested at the same time as those digested for flow cytometry as described above were processed according to standard protocols as described above and reviewed by a urologic pathologist following H&E staining. Areas of cancer, atrophy and benign prostate were identified and scored on each slide. Each of those areas were examined separately for both acute and chronic inflammation. The extent and intensity for each type of inflammation was also examined. If the inflammatory focus was in a limited area across the tissue section it was called focal, otherwise it was called non-focal. The intensity was scored as mild (few scattered cells), moderate (cells forming small group/groups) or severe (large groups of cells or lymphoid follicle formation).

### Differentiation assays

Adipogenic, osteogenic, and chondrogenic differentiation assays were performed using the respective induction medias (Lonza) according to the manufacturer's instructions as previously described [5, 13]. Adipocyte differentiation was determined using the lipid stain Oil Red O (Sigma). Cells were pre-incubated with DHT, R1881, casodex, and/or SB431542 at the respective concentration for 3 days prior to initiation and throughout each induction/maintenance cycle of the assay. For quantification of adipogenic differentiation potential, 0.5 × 10^5^ cells/well were plated in duplicate in a 24-well plate in Rooster High-Performance Media prior to initiation of the assay. Following staining with Oil Red O at the end of the assay, wells were washed 3x with 60% isopropanol and then the dye was solubilized in 100% isopropanol. The absorbance was read at 492 nm with fold change in absorbance calculated over the uninduced negative control. Cultures were tested in 2-3 independent experiments for accurate quantification. Osteoblast differentiation was assayed by staining for calcium deposits using Alizarin Red S (Sigma). Chondrocyte differentiation was evaluated by staining pellets fixed in formalin and paraffin-embedded for glycosaminoglycans with Safranin-O (Sigma).

Smooth muscle differentiation was performed by co-culturing rat or human bone marrow-derived MSCs pre-treated with sodium butyrate [1 mM, (Sigma)] for 48 hrs with rat bladder or human prostate smooth muscle cells, respectively, in a large format (i.e. 6-well) Transwell system [Millicell-PCF 0.4 μm inserts, (Millipore, Billerica, MA)] similar to previously described methods [27]. Rat bone marrow-derived MSCs and bladder smooth muscle cells were obtained from 4-6 wk old Sprague Dawley rats euthanized via CO_2_ overdose in accordance with IACUC-approved protocols. MSCs were aseptically harvested from femurs via flushing the marrow cavity with HBSS using a syringe and culturing marrow in DMEM/F12 supplemented with 10% FBS under standard tissue culture conditions as described above. Bladders were enzymatically digested with collagenase (Sigma) following scraping to remove the epithelial layers and cultured in DMEM supplemented with 10% FBS. Human bone marrow-derived MSCs were obtained as described above and prostate smooth muscle cells were defined as very early passage (p1-2) primary prostate stromal cultures derived from a young organ donor as described above. The use of these very early passage cultures is important because previous work has demonstrated that MSCs are rapidly selected for in primary stromal cultures and progressively become the dominant population within just a few passages [13]. Following 48 hrs of co-culture, the inserts were removed and MSCs harvested from the plate to evaluate smooth muscle differentiation via Western blotting using antibodies to αSMA and SMM.

### Statistical analysis

Correlation of the frequency of MSCs with Gleason score and age were characterized by Spearman rank correlation coefficient. One-way analysis of variance (ANOVA) was used to compare adipogenic differentiation of bone marrow-derived MSCs among the following 5 conditions: a) basal media, b) induction media, c) induction media + 0.3% ethanol (EtOH) vehicle control, d) induction media + 30 nM R1881, and e) induction media + 30 nM DHT. Two-way ANOVA was used to examine the effect of Casodex and SB431542, and the interaction term was tested to assess whether a synergistic effect was observed. A non-significant interaction suggested an additive effect of Casodex and SB431542, and subsequently their main effects were evaluated. Logarithmic transformation was applied to the measures of MSCs to normalize the data. All tests were two-sided and a comparison with a p value of 0.05 or less was considered significant.

Additionally, we would like to acknowledge the Department of Defense Prostate Cancer Research Program, Award No W81XWH-10-2-0056 and W81XWH-10-2-0046 Prostate Cancer Biorepository Network (PCBN), the NIH-Prostate SPORE Grant Pathology Core and Biostatistics Core (P50 CA058236), the Flow Cytometry core, and the Tissue Services Core supported by the SKCCC CCSG (P30 CA006973) for their services and assistance, in addition to acknowledging the use of tissues procured by the National Disease Research Interchange (NDRI) with support from NIH grant 2 U42 OD011158.

## SUPPLEMENTARY MATERIALS TABLES


