# Supplementary material for: Mesenchymal stem cell infiltration during neoplastic transformation of the human prostate
Source: Oncotarget. 2017 Apr 21;8(29):46710–27. doi: 10.18632/oncotarget.17362 (PMC5564518; doi:10.18632/oncotarget.17362)
Supplement: Supplementary file 1 [file oncotarget-08-46710-s001.pdf]

Mesenchymal stem cell infiltration during neoplastic transformation of the human prostate

Supplementary Material

Supplementary Table 1: Androgen does not significantly suppress adipogenesis in human bone marrow-derived MSCs.

Induced	EtOH (0.3%)	R1881 (30 nM)	DHT (30 nM)	Comparison	p-value
+	-	-	-	vs. Uninduced only	$p < 0.0001$
+	+	-	-		$p < 0.0001$
+	-	+	-		$p < 0.0001$
+	-	-	+		$p < 0.0001$
+	+	-	-	vs. Induced only	$p = 0.31$
+	-	+	-		$p = 0.14$
+	-	-	+		$= 0.39$

A significant difference was observed among the five groups overall ($p < 0.0001$). Individual pair-wise comparisons confirmed each treatment group was significantly different compared to the uninduced group. However, no significant difference was detected among the four induced groups overall ($p = 0.5$) or in individual pair-wise comparisons.

Supplementary Table 2: TGF-β inhibition via SB431542 enhances adipogenic differentiation potential of bone marrow- and prostate cancer-derived MSCs with additive effects observed in the context of AR inhibition via Casodex.

Induced	Casodex (20 μM)	SB431542 (5 μM)	Comparison	BM	PCa	NP	FP
+	-	-	vs. Uninduced only	$p < 0.0001$	$p = 0.04$	$p = 0.09$	$p = 0.47$
+	+	-		$p < 0.0001$	$p = 0.02$	$p = 0.08$	$p = 0.59$
+	-	+		$p < 0.0001$	$p = 0.001$	$p = 0.06$	$p = 0.12$
+	+	+		$p < 0.0001$	$p = 0.0003$	$p = 0.07$	$p = 0.55$
+	+	-	vs Induced only	$p = 0.18$	$p = 0.67$	$p = 0.89$	$p = 0.87$
+	-	+		$p = 0.0002$	$p = 0.05$	$p = 0.44$	$p = 0.45$
+	+	+		$p < 0.0001$	$p = 0.01$	$p = 0.61$	$p = 0.91$
+	+	+	vs. Induced + Casodex	$p = 0.07$	$p = 0.03$	$p = 0.72$	$p = 0.96$
+	+	+	vs. Induced + SB431542	$p = 0.78$	$p = 0.55$	$p = 0.79$	$p = 0.39$

A significant difference was detected overall among the five treatment groups in the bone marrow ($p < 0.0001$) and prostate cancer samples ($p = 0.002$), but not the normal ($p = 0.25$) and fetal ($p = 0.62$) prostate samples. Individual pair-wise comparisons confirmed each treatment group was significantly different compared to the uninduced group in the bone marrow and prostate cancer samples, but not normal and fetal prostate samples. A significant difference was also detected overall among the 4 induced groups in the bone marrow ($p = 0.0001$) and prostate cancer ($p = 0.03$) samples, but not the normal ($p = 0.86$) and fetal ($p = 0.77$) prostate samples. Individual pair-wise comparisons confirmed SB431542 alone or in combination with Casodex significantly enhanced adipogenesis in the bone marrow and prostate cancer groups, but not in normal and fetal prostate samples.

Supplementary Table 3: The effect of TGF-β inhibition via SB4315412 and AR inhibition via Casodex on adipogenesis did not reach statistical significance for synergy.

	BM	PCa	NP	FP
Interaction of Casodex and SB431542	$p = 0.45$	$p = 0.9$	$p = 0.77$	$p = 0.62$

Supplementary Table 4: There is an additive effect between SB4315412 and Casodex on adipogenesis in the bone marrow and prostate cancer, but not normal and fetal prostate samples.

	BM	PCa	NP	FP
Casodex + vs. Casodex -	$p = 0.23$	$p = 0.47$	$p = 0.92$	$p = 0.44$
SB431542 + vs. SB431542 -	$p < 0.0001$	$p = 0.003$	$p = 0.39$	$p = 0.55$
